# Supplementary material for: Relationship between emotional labor and sense of career success among community nurses in China, Beijing: A cross-sectional study based on latent class analysis
Source: PLoS One. 2022 May 5;17(5):e0268188. doi: 10.1371/journal.pone.0268188 (PMC9071128; doi:10.1371/journal.pone.0268188)
Supplement: S1 File — (DOCX) [file pone.0268188.s001.docx]

STROBE Statement—checklist of items that should be included in reports of observational studies

|  | Item No. | Recommendation | Page  No. | Relevant text from manuscript |
| --- | --- | --- | --- | --- |
| **Title and abstract** | 1 | (*a*) Indicate the study’s design with a commonly used term in the title or the abstract | 1 | Relationship between emotional labor and sense of career success among community nurses in China, Beijing: a cross-sectional study based on latent class analysis |
|  |  | (*b*) Provide in the abstract an informative and balanced summary of what was done and what was found | 2 | A total of 385 community nurses from Beijing participated in this investigation. Latent class analysis was used to identify meaningful subgroups of participants, and analysis of variance was used to analyze relationships between emotional labor classes and the sense of career success.  Emotional labor among community nursing staff in China was divided into three latent classes: active (n=90, 25.6%), apathetic (n=65, 18.5%), and moderate (n=197, 55.9%). The active emotional labor classes had significantly higher career success (p<0.05). The “gaining recognition” dimension showed significant differences across the three classes. |
| Introduction | | | |  |
| Background/rationale | 2 | Explain the scientific background and rationale for the investigation being reported | 3-4 | The concept of community nursing was first put forward in 1970, and described as the combination of public health and nursing work. The district or community nurse is central to the healthcare of community residents. Currently, community nursing work in China covers basic therapeutic care (70%) and public health services (30%), such as inpatient and outpatient care, preventive healthcare, maternal and child healthcare, chronic disease management, health education, and family nursing. The complexity of the community nursing working environment and the diversity of services creates higher requirements in terms of the knowledge and abilities of community nurses. To meet the requirements of their job, community nurses need to provide high-quality mental and physical healthcare services, and also need to maintain the necessary emotional communication with service recipients to achieve performance satisfaction. To understand patients’ experiences and support relationships with patients, relatives, and colleagues, community nurses use emotional labor and control their emotional expressions. Emotional labor is the third kind of labor after physical and mental labor, and refers to the process of psychological adjustment necessary to express the emotions expected by an organization. Nurses should know and control the process required for managing their emotions and expressions. Excessive emotional labor can cause mental health problems, musculoskeletal symptoms, cardiovascular disease, and other physical health problems. Although emotional labor is an important part of nursing practice, it is usually invisible, neglected and under estimated. And even literature shows that emotional labor exists across a broad spectrum of nursing work, only few researches on conditions and related factors of community nurses’ emotional labor is available, and this topic remains to be investigated.  With the expansion of the scope of community nursing and improvement in quality requirements, the psychological pressure on community nurses has increased, along with issues associated with professional satisfaction and career success. Career success refers to the gradual accumulation or acquisition of work-related achievements and positive feelings by individuals throughout their work experience, including remuneration, promotion, recognition, and acceptance. A previous study among nurses indicated that the process of applying emotional labor aimed to achieve organizational goals and improve nurses’ salary, promotion opportunities, and recognition. Previous studies have shown that emotional labor strategies have significant predictive effects on job stress, job satisfaction, and subjective job performance. Researchers also found that nurses’ career success was mainly affected by emotional labor. How about the relationship between career success and emotional labor in community nurses is need to be explored.  Emotional labor is the multi-level and dynamic essence of emotional regulation, and includes surface acting (e.g., concealing negative emotion with a fake smile), deep acting (e.g., taking a positive and optimistic approach to eliminate negative emotions and conform to the requirements of the organization), and natural expression dimensions (e.g., the true expression of one’s emotions in accordance with work requirements). The effect of emotional labor is the result of the mixed effect of these dimensions. Previous research on emotional labor in nursing mainly centered on the degree of total and each dimension of emotional labor or their relationship with other occupational factors, and the results did not clarify the specific situation and characteristics of emotional labor for an individual. Therefore, even if the scores for emotional labor measurement were consistent, internal heterogeneity could not be determined. To solve this problem, it is necessary to use an individual-centered research method to analyze the heterogeneity of emotional labor among community nurses. Latent class analysis (LCA) focuses on the heterogeneity of individuals, and the latent class classification of individuals can be determined according to the response pattern of an individual in the detection questions; this means the number and proportion in each category can be clarified [32]. Therefore, the level and internal characteristics of emotional labor of community nurses are a research issue that needs further discussion. |
| Objectives | 3 | State specific objectives, including any prespecified hypotheses | 4 | Analysis of the potential categories of emotional labor among community nurses (from surface variable analysis to internal characteristic analysis) and the relationship between the level of emotional labor and the sense of career success were taken based on LCA. The results may be valuable for improving the emotional labor among community nurses, which may provide scientific data for improving the quality of community nursing services and promoting career success and team stability. |
| Methods | | | |  |
| Study design | 4 | Present key elements of study design early in the paper | 5 | This study was a cross-sectional survey. |
| Setting | 5 | Describe the setting, locations, and relevant dates, including periods of recruitment, exposure, follow-up, and data collection | 5-6 | The survey was conducted during the period from Sep 10 to Sep 30, 2020.We recruited community nurses from 11 community health service centers in four districts in Beijing using convenience sampling. |
| Participants | 6 | (*a*) *Cohort study*—Give the eligibility criteria, and the sources and methods of selection of participants. Describe methods of follow-up  *Case-control study*—Give the eligibility criteria, and the sources and methods of case ascertainment and control selection. Give the rationale for the choice of cases and controls  *Cross-sectional study*—Give the eligibility criteria, and the sources and methods of selection of participants |  | The inclusion criteria were nursing staff: (1) with a nurse’s certificate; (2) pass the community nurse on-the-job training prescribed by health administrative department of province or city; (3) that had worked in the participating community health institutions for ≥1 year and (4) that provided informed consent for voluntary participation. Exclusion criteria were: (1) nurses that had returned to work after sick or casual leave <1 year previously; (2) nursing managers; (3) student nurses; and (4) nursing staff who were going to retire within 3 months.  Nurses in selected communities who met the inclusion criteria were all participated in our study. The research described in this paper meets the ethical guidelines of the ethics committee of the Peking University Health Science Center. And it was approved by the ethics committee of the Biomedical Ethics Committee of Peking University (ref.no. IRB00001052-20052). Nursing department of each selected community institutions assisted to organize the nurses who participated in the survey to fill in the questionnaire together. Before the survey, members of the research team explained the purpose and process of the study to potential participants and individualized data were kept confidential. They were informed that the investigation process was anonymous, and they were free to withdraw at any time. If they wished to participate, an informed consent form was signed and they were included in this study. The members of the investigation group then sent out questionnaires to participants with instructions on completing the survey using unified guiding language. Participants completed the questionnaire by themselves, and members of the investigation team checked whether the questionnaire was complete. The questionnaires were completed in an office room environment. |
|  |  | (*b*) *Cohort study*—For matched studies, give matching criteria and number of exposed and unexposed  *Case-control study*—For matched studies, give matching criteria and the number of controls per case |  |  |
| Variables | 7 | Clearly define all outcomes, exposures, predictors, potential confounders, and effect modifiers. Give diagnostic criteria, if applicable | 6 | The research questionnaire comprised three parts: (a) general information questionnaire; (b) the Emotional Labor Scale (ELS); and (c) the Career Success Scale (CSS). |
| Data sources/ measurement | 8* | For each variable of interest, give sources of data and details of methods of assessment (measurement). Describe comparability of assessment methods if there is more than one group | *6* | The general information questionnaire included 31 items such as gender, age, marital status, education level, work years, daily working hours, position, and continuing study. ELS was developed by Diefendorff and revised by Yin. The three-dimensional sale comprises 14 items and is used to measure core characteristics of emotional labor: surface acting (seven items), deep acting (four items), and natural expression (three items). The CSS was developed from the career satisfaction scale. A Chinese version was developed by Li Ya. The 21 questionnaire items are divided into four dimensions: career development (five questions), freedom and happiness (five questions), gain recognition (six questions), and international network (five questions). Responses are on a five-point Likert scale (1=strongly disagree; 5=strongly agree), with a total possible score of 105. |
| Bias | 9 | Describe any efforts to address potential sources of bias |  |  |
| Study size | 10 | Explain how the study size was arrived at | 5 | The sample size was based on the basic proportion of the scale items, with one item corresponding to10 samples. The required sample size for this study was 350. Considering the possibility of a 10% sample loss (n=35), the final sample size for this study was calculated at 385 community nurses. |

Continued on next page

| Quantitative variables | 11 | Explain how quantitative variables were handled in the analyses. If applicable, describe which groupings were chosen and why | 6 | Five-point Likert scale (1=strongly disagree; 5=strongly agree) |
| --- | --- | --- | --- | --- |
| Statistical methods | 12 | (*a*) Describe all statistical methods, including those used to control for confounding | 7 | Data were analyzed using SPSS version 20.0. Count data were described by frequency composition ratio, and data with a normal distribution were described by mean±standard deviation. Mplus version 7.0 was used to analyze the potential categories of emotional labor. Mplus software was developed by Linda Muthen and Bengt Muthen in 1998 and It is the most popular and powerful latent variable analysis software at present . LCA model fit test indices included: Akaike information criterion (AIC), Bayesian information criterion (BIC), accommodated BIC, entropy value, Lo-Mendell Rubin (LMR) test, and bootstrap likelihood ratio test (BLRT) . Smaller AIC and BIC values indicate a better simulation fit. Entropy ranges from 0 to 1, and represent the accuracy of model classification; entropy ≥0.8 indicates that the classification accuracy exceeds 90%. In addition, the fit differences of potential category models can also be determined by LMR and BLRT, and p-values <0.05 indicated the differences were statistically significant. The establishment of the optimal model considered the adaptation index and existing research and the interpretability of data results. After the optimal model was chosen, the differences in sociodemographic variables in each class allocation were obtained using chi-squared tests. Analysis of variance (ANOVA) was used to analyze the influence of a sense of career success on classification of emotional labor. P-values <0.05 were considered statistically significant. |
|  |  | (*b*) Describe any methods used to examine subgroups and interactions |  |  |
|  |  | (*c*) Explain how missing data were addressed |  |  |
|  |  | (*d*) *Cohort study*—If applicable, explain how loss to follow-up was addressed  *Case-control study*—If applicable, explain how matching of cases and controls was addressed  *Cross-sectional study*—If applicable, describe analytical methods taking account of sampling strategy |  |  |
|  |  | (*e*) Describe any sensitivity analyses |  |  |
| Results | | | | |
| Participants | 13* | (a) Report numbers of individuals at each stage of study—eg numbers potentially eligible, examined for eligibility, confirmed eligible, included in the study, completing follow-up, and analysed | 7 | A total of 382 questionnaires were completed anonymously and 352 valid questionnaires were recovered (effective recovery rate: 96.8%). |
|  |  | (b) Give reasons for non-participation at each stage |  |  |
|  |  | (c) Consider use of a flow diagram |  |  |
| Descriptive data | 14* | (a) Give characteristics of study participants (eg demographic, clinical, social) and information on exposures and potential confounders | 7-8 | Participants’ mean age was 36.93±9.29 years and most were female (98.9%). Most participants (49.1%) had an associate degree, 45.2% has a bachelor’s degree or above, and 5.7% had a technical secondary school or below qualification. The average daily work duration was 8.11±0.68 hours, 20.5% (n=72) of participants participated in continuing education, and 4.3% (n=15) were engaged in scientific research. Community nursing work involved various roles such as nursing operations, family visits, chronic disease management, health education, prevention, and healthcare services. |
|  |  | (b) Indicate number of participants with missing data for each variable of interest |  |  |
|  |  | (c) *Cohort study*—Summarise follow-up time (eg, average and total amount) |  |  |
| Outcome data | 15* | *Cohort study*—Report numbers of outcome events or summary measures over time |  |  |
|  |  | *Case-control study—*Report numbers in each exposure category, or summary measures of exposure |  |  |
|  |  | *Cross-sectional study—*Report numbers of outcome events or summary measures | *9-14* | *4* |
| Main results | 16 | (*a*) Give unadjusted estimates and, if applicable, confounder-adjusted estimates and their precision (eg, 95% confidence interval). Make clear which confounders were adjusted for and why they were included |  |  |
|  |  | (*b*) Report category boundaries when continuous variables were categorized |  |  |
|  |  | (*c*) If relevant, consider translating estimates of relative risk into absolute risk for a meaningful time period |  |  |

Continued on next page

| Other analyses | 17 | Report other analyses done—eg analyses of subgroups and interactions, and sensitivity analyses |  |  |
| --- | --- | --- | --- | --- |
| Discussion | | | | |
| Key results | 18 | Summarise key results with reference to study objectives | 14-17 | The LCA results identified a 3-class model: class 1=active group (n=90, 25.6%), class 2=apathetic group (n=65, 18.5%), and class 3=moderate group (n=197, 55.9%). The total mean emotional labor score was 3.51±0.49, which was higher than the medium critical value of 3.00 using the Likert scale 5-point scoring method. Active class of community nurses had the highest sense of career success, followed by the moderate class and then the apathetic group. |
| Limitations | 19 | Discuss limitations of the study, taking into account sources of potential bias or imprecision. Discuss both direction and magnitude of any potential bias | 17 | A limitation of this study was that it was a cross-sectional study rather than a longitudinal study. In addition, the sample size of this study was not large and widely enough, participants were nurses from 11 health service centers in four urban areas of Beijing, therefore, the representativeness and conclusions of its study were limited, and the results cannot represent all Chinese nurses. Further research may need to focus on nurses from national communities. |
| Interpretation | 20 | Give a cautious overall interpretation of results considering objectives, limitations, multiplicity of analyses, results from similar studies, and other relevant evidence |  |  |
| Generalisability | 21 | Discuss the generalisability (external validity) of the study results | 18 | The emotional labor of community nurses in China can be divided into three classes: positive, apathetic, and moderate. Managers could implement a variety of measures to strengthen interventions for employees’ emotional labor that are targeted to incentive mechanisms, which will improve nurses’ sense of career success. |
| Other information | |  | | |
| Funding | 22 | Give the source of funding and the role of the funders for the present study and, if applicable, for the original study on which the present article is based |  |  |

*Give information separately for cases and controls in case-control studies and, if applicable, for exposed and unexposed groups in cohort and cross-sectional studies.

**Note:** An Explanation and Elaboration article discusses each checklist item and gives methodological background and published examples of transparent reporting. The STROBE checklist is best used in conjunction with this article (freely available on the Web sites of PLoS Medicine at http://www.plosmedicine.org/, Annals of Internal Medicine at http://www.annals.org/, and Epidemiology at http://www.epidem.com/). Information on the STROBE Initiative is available at www.strobe-statement.org.
