# Supplementary material for: Relationship between emotional labor and sense of career success among community nurses in China, Beijing: A cross-sectional study based on latent class analysis
Source: PLoS One. 2022 May 5;17(5):e0268188. doi: 10.1371/journal.pone.0268188 (PMC9071128; doi:10.1371/journal.pone.0268188)
Supplement: Attachment — Submitted filename: Response to Reviewers.docx [file pone.0268188.s002.docx]

**Response to Reviewers**

Dear Reviewers,

On behalf of my co-authors, we thank you very much for giving us an opportunity to revise our manuscript. We appreciate reviewers very much for their positive and constructive comments and suggestions on our manuscript entitled “Relationship between emotional labor and sense of career success among community nurses in China, Beijing: a study based on latent class analysis” (PONE-D-21-54188). The revised version of our manuscript has been modified according to the reviewers’ suggestions.

The following is a point-to-point response to the editors’ and reviewers’ comments.

*Reviewer #1: Comments
 Background:* *You should clearly state the research problem (Much better). The author starts with the research objective.*

The research problem of our study is “The potential categories of community nurses’ emotional labor and related factors”. We made it clear in the Introduction part.

*Why this topic*

We chose emotional labor as the study topic because excessive emotional labor can cause physical and mental health problems, job stress, burnout, et al. Although emotional labor is an important part of nursing practice, it is usually invisible, neglected and under estimated. So, this topic remains to be investigated. We made it clear in the Introduction part.

*What is the problem*

The problem is “what is the level and the internal characteristics of emotional labor of community nurses and its related factors”. We made it clear in the Introduction part.

*Method:* *Better to indicate your research design*

We added research design in the Method part of the manuscript, including inclusion criteria, sampling methods and distributing and retrieving questionnaires procedure, et al.

*Conclusion:* *Mention the major finding, then continue with your recommendation.*

We abbreviated the Conclusion part and leaving the major finding of the study.

*Result
Associate degree not clear try to clarify.*

We revised the associate degree referred to the literature that used the same grouping method, and revised the Table 1. Hoping more clearly this time.

*Technical secondary school or below what does this mean?*

The meaning of “the technical secondary school or below” means nurses graduated from the secondary vocational school.

*The participants were community nurses. Explain how community nurses were recruited in your area somewhere in the methodology OR what is community nurses?*

We added related information in the Method part to explain the criteria of the community nursing in China and how we recruited them.

Community nurse in China are nurses (with the nurse’s certificate) who pass the community nurse on-the-job training prescribed by health administrative department of province or city and working in the community health institution.

Based on the principle of convenient cluster sampling, 11 community health service institutions in Beijing were selected. And community nurses in the 11 community health service institutions were potential participator of the study.

*What is their educational level in your setup?*

The education level of the community nurses in our setup was revised in the Result part: 5.7%graduate from secondary vocational schools, 49.1%participants had an associate degree, 45.2% has a bachelor’s degree or above.

*Discussion and conclusion*
*Too long with unclear explanation. Try to choose major findings and discuss them by comparing them with the literature together with your assumption.*

We rewrote the Discussion part and made it clearer. And only the main findings were retained and we discussed our assumption by comparing with the related literature.

*The manuscript should answer “So what”.*

Yes, we rewrote the Discussion part of the manuscript and emphasized the scientific and practical significance of this study.

*What is the take-home message?*

The discussion section was rewritten to delete the long and irrelevant content, highlight the research focus, and reflect the key information.

*The manuscript has many terminologies which is difficult for readers, so better to have some operational definition.*

We added operation definition or explanations of the terminologies in the revised version and hope it easier to be understood.

*Reviewer #2: Dear authors mine study 'Relationship between emotional labor and sense of career success among community*

*nurses in China, Beijing: a study based on latent class analyses was well done study.*

*Add the caption to the figure on page 5418.*

We added the caption of the Figure in the revised manuscript.
